# Identification of genes from ten oncogenic pathways associated with mortality and disease progression in glioblastoma

**DOI:** 10.3389/fonc.2022.965638

**Published:** 2022-08-10

**Authors:** Myung-Hoon Han, Kyueng-Whan Min, Yung-Kyun Noh, Jae Min Kim, Jin Hwan Cheong, Je Il Ryu, Yu Deok Won, Seong-Ho Koh, Young Mi Park

**Affiliations:** ^1^ Department of Neurosurgery, Hanyang University Guri Hospital, Hanyang University College of Medicine, Guri, South Korea; ^2^ Department of Pathology, Hanyang University Guri Hospital, Hanyang University College of Medicine, Guri, South Korea; ^3^ Department of Computer Science, Hanyang University, Seoul, South Korea; ^4^ School of Computational Sciences, Korea Institute for Advanced Study, Seoul, South Korea; ^5^ Department of Neurology, Hanyang University Guri Hospital, Hanyang University College of Medicine, Guri, South Korea; ^6^ Department of Pediatrics, Gangneung Asan Hospital, Ulsan University College of Medicine, Gangneung-si, South Korea

**Keywords:** glioblastoma multiforme, oncogenic signaling pathways, The Cancer Genome Atlas, survival, gene

## Abstract

Glioblastoma multiforme (GBM) is the most malignant brain tumor with an extremely poor prognosis. The Cancer Genome Atlas (TCGA) database has been used to confirm the roles played by 10 canonical oncogenic signaling pathways in various cancers. The purpose of this study was to evaluate the expression of genes in these 10 canonical oncogenic signaling pathways, which are significantly related to mortality and disease progression in GBM patients. Clinicopathological information and mRNA expression data of 525 patients with GBM were obtained from TCGA database. Gene sets related to the 10 oncogenic signaling pathways were investigated *via* Gene Set Enrichment Analysis. Multivariate Cox regression analysis was performed for all the genes significantly associated with mortality and disease progression for each oncogenic signaling pathway in GBM patients. We found 12 independent genes from the 10 oncogenic signaling pathways that were significantly related to mortality and disease progression in GBM patients. Considering the roles of these 12 significant genes in cancer, we suggest possible mechanisms affecting the prognosis of GBM. We also observed that the expression of 6 of the genes significantly associated with a poor prognosis of GBM, showed negative correlations with CD8+ T-cells in GBM tissue. Using a large-scale open database, we identified 12 genes belonging to 10 well-known oncogenic canonical pathways, which were significantly associated with mortality and disease progression in patients with GBM. We believe that our findings will contribute to a better understanding of the mechanisms underlying the pathophysiology of GBM in the future.

## Introduction

Glioblastoma multiforme (GBM) is the most malignant and common primary brain tumor with an extremely poor prognosis. Despite the variety of modern therapies against GBM, the median survival of GBM patients is approximately 14 to 15 months from the diagnosis (1).

The Cancer Genome Atlas (TCGA) is the world’s largest and richest collection of genomic data, including digital pathologic slides, clinicopathological information, and gene expression and RNA sequencing data (2). TCGA data have confirmed the roles of 10 canonical oncogenic signaling pathways with frequent genetic alterations in cancer (3). The 10 oncogenic signaling pathways are cell cycle, Hippo, Myc, Notch, nuclear factor erythroid 2-related factor 2 (Nrf2), phosphatidylinositol 3-kinase (PI3K), receptor tyrosine kinase (RTK), transforming growth factor beta (TGF-β), p53, and Wnt/β-catenin (3). With the aid of TCGA, we recently showed that dickkopf-3 (DKK3) in the Wnt/β-catenin signaling pathway is associated with an immunosuppressive tumor microenvironment and a poor prognosis in patients with GBM (4). Since Wnt/β-catenin signaling is one of the 10 oncogenic signaling pathways, we expanded the scope of the study to use TCGA data to identify additional genes in the remaining 9 oncogenic signaling pathways that are significantly related to mortality and disease progression in GBM patients.

Therefore, the primary aim of the study was to use TCGA data to identify genes in the 10 canonical oncogenic signaling pathways, which are significantly associated with mortality and disease progression in GBM patients. The secondary objective of the study was to investigate correlations between these genes and between these genes and immunosuppressive conditions in GBM. Further, drug sensitivity screening was also performed in GBM cell lines using the Genomics of Drug Sensitivity in Cancer (GDSC) and the Catalog of Somatic Mutations in Cancer (COSMIC) databases.

## Materials and methods

### Study patients

As we recently reported, the same 525 GBM cases with virtual histopathological slides, clinical information, and mRNA expression data from TCGA were used to conduct this study (https://gdc.cancer.gov/about-data/publications/pancanatlas and https://www.cbioportal.org/) (4). The raw data used in this study are presented in the Supplementary Data 1 file.

Extra informed consent is not essential because the data were all obtained from public TCGA database.

### Gene sets related to the 10 oncogenic signaling pathways

To identify genes significantly associated with disease progression and mortality in GBM, we used the Molecular Signatures Database (MSigDB version 7.5.1) with 32,880 gene sets of the Gene Set Enrichment Analysis (GSEA) (version 4.1.0) (https://www.gsea-msigdb.org/) (5). Gene sets related to the 10 oncogenic signaling pathways were investigated via GSEA. The gene sets in the 10 canonical signaling pathways used for the analysis are from the following signaling pathways (1): cell cycle (standard name, KEGG_CELL_CYCLE; systematic name, M7963; when matching with TCGA database, 112 genes out of 125 were available for analysis) (2), Hippo signaling (standard name, GOBP_HIPPO_SIGNALING; systematic name, M11445; 29 genes out of 40 were available for analysis) (3), Myc signaling (standard name, PID_MYC_PATHWAY; systematic name, M139; 23 genes out of 25 were available for analysis) (4), Notch signaling (standard name, KEGG_NOTCH_SIGNALING_PATHWAY; systematic name, M7946; 36 genes out of 47 were available for analysis) (5), Nrf2 signaling (standard name, WP_NRF2_PATHWAY; systematic name, M39454; 106 genes out of 145 were available for analysis) (6), PI3K signaling (standard name, HALLMARK_PI3K_AKT_MTOR_SIGNALING; systematic name, M5923; 97 genes out of 105 were available for analysis) (7), RTK signaling (standard name, KEGG_ERBB_SIGNALING_PATHWAY; systematic name, M12467; 80 genes out of 87 were available for analysis) (8), TGF-β signaling (standard name, KEGG_TGF_BETA_SIGNALING_PATHWAY; systematic name, M2642; 79 genes out of 86 were available for analysis) (9), p53 signaling (standard name, KEGG_P53_SIGNALING_PATHWAY; systematic name, M6370; 59 genes out of 68 were available for analysis), and (10) Wnt/β-catenin signaling (standard name, ST_WNT_BETA_CATENIN_PATHWAY; systematic name, M17761; 31 genes out of 34 were available for analysis). We present raw data related to the mRNA levels of all gene sets in GBM tissues for each oncogenic signaling pathway used in the analysis in the **Supplementary Data File 2**.

### In silico flow cytometry

As described previously, the immune cell composition of GBM tissues in our study patients was identified using CIBERSORT (https://cibersort.stanford.edu), which is a computational program to decipher the immune cell-type fractions based on a validated leukocyte gene signature matrix containing 547 genes and 22 human immune cell subpopulations (4, 6). We entered the gene expression profiles of GBM tissues from TCGA into CIBERSORT for analysis, and the algorithm was run using the LM22 signature matrix at 100 permutations. There is a positive correlation between elevated CD8+ T cell counts in the tumor microenvironment and a good prognosis in cancer (7). In addition, because CD8+ T cells are considered major drivers of antitumor immunity, we included CD8+ T cells in our examination of the relationship between gene expression and the degree of antitumor immunity in the GBM microenvironment in this study (8).

### Data extraction from the GDSC and COSMIC databases

We performed drug screening using datasets from the GDSC and COSMIC databases, which are large-scale cancer cell line and drug response databases containing data from 1,796 cancer cell lines and 565 compounds, respectively (9, 10). We examined the gene expression in 49 GBM cell lines (A172, AM-38, Becker, CAS-1, CCF-STTG1, D-247MG, D-263MG, D-336MG, D-392MG, D-423MG, D-502MG, D-542MG, D-566MG, DBTRG-05MG, DK-MG, GAMG, GB-1, GI-1, GMS-10, H4, KALS-1, KINGS-1, KNS-42, KNS-81-FD, KS-1, LN-18, LN-229, LN-405, LNZTA3WT4, M059J, MOG-G-CCM, MOG-G-UVW, NMC-G1, no-10, no-11, SF126, SF268, SF295, SF539, SK-MG-1, SNB75, SW1088, SW1783, T98G, U-118-MG, U-87-MG, U251, YH-13, YKG-1). The responses of 49 GBM cell lines to 316 drugs were measured and reported as the natural log half-maximal inhibitory concentration [ln(IC50)] value. A drug was defined as being effective when a negative correlation was observed between gene expression and the ln(IC50) values in 49 GBM cell lines with a p value < 0.05 (11, 12).

### Statistical analysis

Pearson’s correlation coefficients and significance levels (p-values) were estimated to examine the associations between the mRNA expression of the above-mentioned 12 genes from the 10 oncogenic signaling pathways and between the gene expression of these 12 genes and CD8+ T cell fractions using R with clustering technique (R code: corrplot, M, order = “hclust”, p.mat = p_mat, sig.level = 0.01, method = “square”). In addition, Pearson’s correlation analysis was also performed to identify the correlation between gene expression and ln(IC50) values for various antitumor agents in 49 GBM cell lines.

We estimated overall survival (OS) and progression-free survival (PFS) using Kaplan–Meier analysis for all genes related to each oncogenic signaling pathway based on the gene expression tertiles (tertile 1 = low expression; tertile 2 = moderate expression; tertile 3 = high expression) (4). First, we identified all genes for each oncogenic signaling pathway that were significantly related to OS and PFS of the Kaplan–Meier analysis. We then performed multivariate Cox regression analysis for only those genes to identify independent genes associated with OS and PFS in GBM patients.

Linear regression analysis using R was performed to visualize associations between age and gene expressions of 12 selected genes and gene expression and ln(IC50) values for antitumor agents in 49 GBM cell lines. Regression lines and a line determined by locally weighted scatter plot smoothing (LOWESS) were used to visualize the association between age and expression of the 12 selected genes (13).

A p-value < 0.05 was considered statistically significant. All statistical analyses were performed using R software version 4.1.2 and SPSS for Windows version 24.0 (IBM, Chicago, IL).

## Results

### Characteristics of patients

Altogether, 525 patients with GBM from TCGA database were enrolled in the study. The mean patient age at diagnosis was 57.7 years, and 39.0% of patients were female. A total of 435 (82.9%) patients had received radiation treatment, and 39.0% of the patients received adjuvant chemotherapy and/or immunotherapy. The mean CD8+ T cell fraction in GBM tissues was 0.022; further detailed information is shown in **Table 1**.

**Table 1:** Clinical characteristics of the study patients with GBM.

Characteristics	Total
Number	525
Sex, female, n (%)	205 (39.0)
Age at diagnosis of GBM, mean ± SD, y	57.7 ± 14.6
Time duration between GBM diagnosis and death (days), mean ± SD	508.9 ± 539.4
Time duration between GBM diagnosis and disease progression (days), mean ± SD	307.0 ± 391.0
Karnofsky performance scale score, median (IQR)	80.0 (70.0–80.0)
Missing data, n (%)	133 (25.3)
Radiation treatment, n (%)	
Yes	435 (82.9)
No	70 (13.3)
Missing data	20 (3.8)
Adjuvant chemotherapy and/or immunotherapy, n (%)	
Yes	205 (39.0)
No	2 (0.4)
Missing data	318 (60.6)
History of prior glioma, n (%)	15 (2.9)
Immune cells (CIBERSORT fraction), mean ± SD	
CD8+ T cells	0.022 ± 0.039

GBM, glioblastoma multiforme; SD, standard deviation; IQR, interquartile range.

### Identification of genes from the 10 oncogenic signaling pathways associated with both OS and PFS in GBM patients

**Figure 1:**
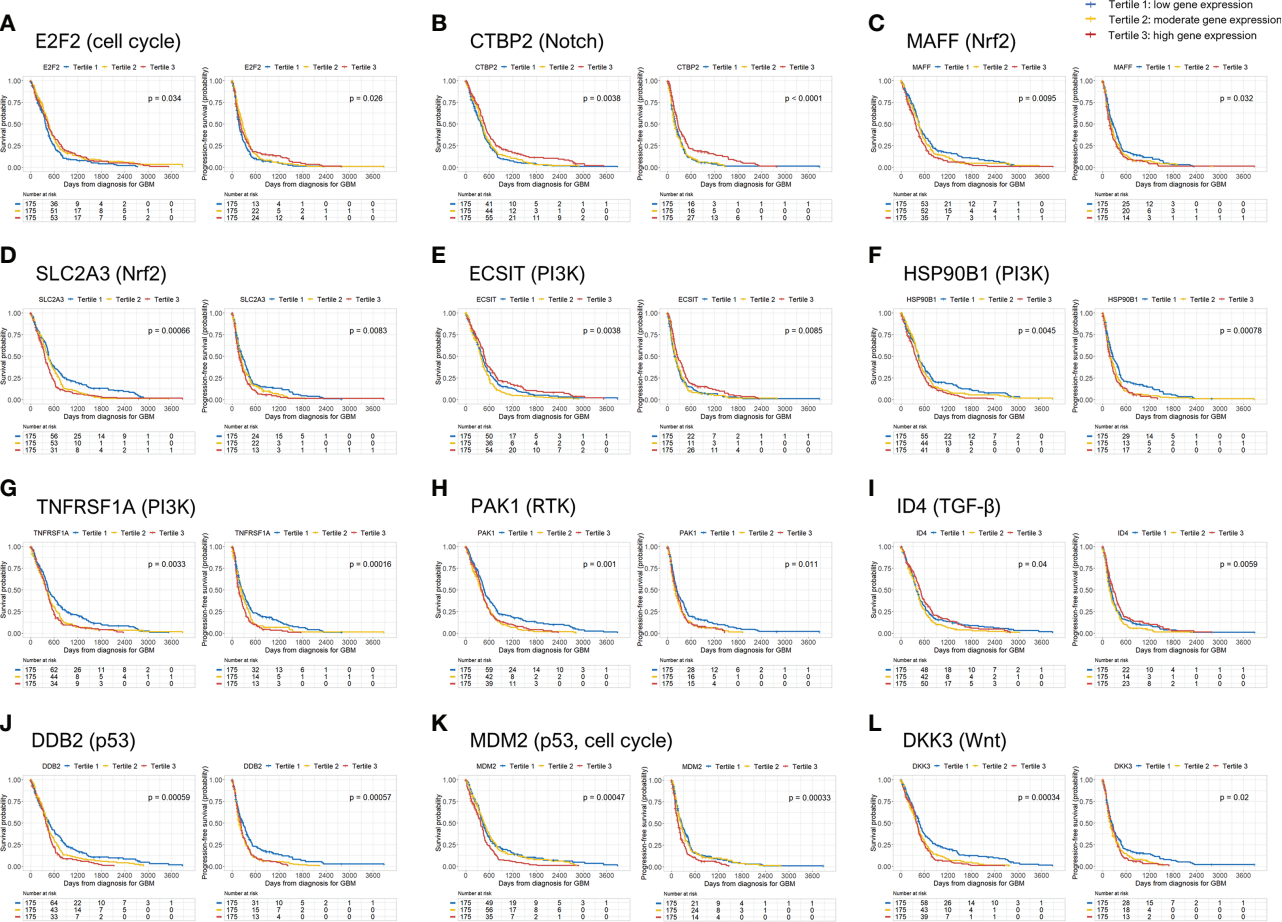
Overall survival (OS) and progression-free survival (PFS) rates according to 12 genes significantly associated with mortality and disease progression in GBM patients. GBM, glioblastoma.

**Table 2** shows all the genes, classified by each 10 oncogenic signaling pathway, that were significantly associated with both OS and PFS in Kaplan-Meier survival analysis with log-rank test. When we performed multivariate Cox regression analysis, we found 12 such independent genes from the 10 oncogenic signaling pathways. The identified 12 significant genes were (1): E2F transcription factor 2 (E2F2) (tertile 2 vs. tertile 1, OS: hazard ratio (HR), 0.74; p = 0.032; PFS: HR, 0.75; p = 0.034) from the cell cycle signaling pathway (2), C-terminal binding protein 2 (CTBP2) (tertile 3 vs. tertile 1, OS: HR, 0.69; p = 0.010; PFS: HR, 0.74; p = 0.032) from the Notch signaling pathway (3), MAF bZIP transcription factor F (MAFF) (tertile 1 vs. tertile 3, OS: HR, 0.74; p = 0.036; PFS: HR, 0.64; p = 0.002) from the Nrf2 signaling pathway (4), solute carrier family 2 member 3 (SLC2A3) (tertile 1 vs. tertile 3, OS: HR, 0.75; p = 0.037; PFS: HR, 0.71; p = 0.012) from the Nrf2 signaling pathway (5), evolutionarily conserved signaling intermediate in Toll pathways (ECSIT) (tertile 2 vs. tertile 3, OS: HR, 1.69; p < 0.001; PFS: HR, 1.41; p = 0.016) from the PI3K signaling pathway (6), heat shock protein 90 kDa beta member 1 (HSP90B1) (tertile 1 vs. tertile 3, OS: HR, 0.74; p < 0.034; PFS: HR, 0.57; p < 0.001) from the PI3K signaling pathway (7), tumor necrosis factor receptor superfamily member 1A (TNFRSF1A) (tertile 1 vs. tertile 3, OS: HR, 0.75; p < 0.041; PFS: HR, 0.63; p = 0.001) from the PI3K signaling pathway (8), p21 activated kinase 1 (PAK1) (tertile 1 vs. tertile 3, OS: HR, 0.60; p = 0.001; PFS: HR, 0.75; p = 0.044) from the RTK signaling pathway (9), inhibitor of DNA binding 4 (ID4) (tertile 2 vs. tertile 1, OS: HR, 1.53; p = 0.002; PFS: HR, 1.41; p = 0.009) from the TGF-β signaling pathway (10), damage-specific DNA-binding protein 2 (DDB2) (tertile 1 vs. tertile 3, OS: HR, 0.73; p = 0.031; PFS: HR, 0.72; p = 0.024) from the P53 signaling pathway (11), mouse double minute 2 homolog (MDM2) (tertile 1 vs. tertile 3, OS: HR, 0.68; p = 0.006; PFS: HR, 0.63; p = 0.001) from the p53 signaling and cell cycle pathways, and (12) dickkopf-3 (DKK3) (tertile 1 vs. tertile 3, OS: HR, 0.73; p = 0.031; PFS: HR, 0.75; p = 0.044) from the Wnt/β-catenin signaling pathway (**Table 2**). When we examined the relationship between age and the expression of the 12 selected genes, we found that the expression of E2F2 and CTBP2 decreased slightly with age in GBM patients (**Supplementary Figure 1**). In contrast, the expression of MAFF, SLC2A3, TNFRSF1A, DDB2, and DKK3 gradually increased with age (**Supplementary Figures 1, 2**). However, as shown above, when we adjusted all variables including age in the multivariate analysis, the significant associations between the 12 selected genes and both OS and PFS were maintained in patients with GBM. In addition, we identified the differences between the radiation treatment population and the adjuvant chemotherapy and/or immunotherapy population on the basis of expression of the 12 selected genes in patients with GBM (Supplementary **Table 1**). There were significant differences in the expression of MAFF and ECSIT in the adjuvant chemotherapy and/or immunotherapy populations. Moreover, even when factors like radiation treatment and chemotherapy were adjusted, MAFF and ECSIT still showed significant correlations with both OS and PFS in GBM patients. We also present the Kaplan-Meier survival curves for OS and PFS in GBM patients according to the tertile groups of gene expression of the 12 significant genes in **Figure 1**.

**Table 2:** Multivariate Cox analyses of genes significantly associated with overall survival and progression-free survival in GBM patients related to 10 oncogenic signaling pathways.

	Overall survival			Progression-free survival		
	Log-rank test (Kaplan-Meier analysis)	Multivariate Cox regression analysis*		Log-rank test (Kaplan-Meier analysis	Multivariate Cox regression analysis*	
**Oncogenic signaling pathways**	p	HR (95% CI)	p	p	HR (95% CI)	p
**1. Cell cycle signaling (112 genes were analyzed)**						
CDC14A (tertile 3 vs. tertile 1)	0.009	0.90 (0.68–1.18)	0.441	<0.001	0.67 (0.51–0.88)	0.004
CDKN2A (tertile 3 vs. tertile 1)	0.002	0.79 (0.60–1.04)	0.093	0.015	0.78 (0.59–1.02)	0.072
**E2F2 (tertile 2 vs. tertile 1)**	**0.034**	**0.74 (0.56–0.97)**	**0.032**	**0.026**	**0.75 (0.57–0.98)**	**0.034**
**(tertile 2 vs. tertile 3)**	**0.034**	**0.75 (0.57–0.98)**	**0.036**	0.026	0.84 (0.64–1.10)	0.200
MAD2L1 (tertile 3 vs. tertile 1)	0.020	0.75 (0.57–0.99)	0.043	0.037	0.89 (0.68–1.17)	0.407
**MDM2 (tertile 1 vs. tertile 3)**	**<0.001**	**0.68 (0.52–0.90)**	**0.006**	**<0.001**	**0.63 (0.48–0.83)**	**0.001**
SMC3 (tertile 3 vs. tertile 1)	0.013	0.72 (0.55–0.95)	0.021	0.011	0.83 (0.63–1.09)	0.172
ZBTB17 (tertile 2 vs. tertile 1)	0.021	0.69 (0.53–0.91)	0.007	0.013	0.79 (0.61–1.03)	0.083
**2. Hippo signaling (29 genes were analyzed)**						
AMOT (tertile 3 vs. tertile 1)	0.016	0.80 (0.61–1.05)	0.106	0.048	0.81 (0.62–1.06)	0.122
DLG5 (tertile 3 vs. tertile 1)	0.006	0.67 (0.51–0.89)	0.005	0.019	0.86 (0.66–1.13)	0.283
TIAL1 (tertile 3 vs. tertile 1)	0.008	0.72 (0.55–0.95)	0.021	0.026	0.87 (0.67–1.13)	0.300
WWTR1 (tertile 1 vs. tertile 3)	0.003	0.87 (0.66–1.15)	0.336	0.006	0.69 (0.52–0.91)	0.009
**3. Myc signaling (23 genes were analyzed)**						
CDKN2A (tertile 3 vs. tertile 1)	0.002	0.79 (0.60–1.04)	0.093	0.015	0.78 (0.59–1.02)	0.072
ZBTB17 (tertile 2 vs. tertile 1)	0.021	0.69 (0.53–0.91)	0.007	0.013	0.79 (0.61–1.03)	0.083
**4. Notch signaling (36 genes were analyzed)**						
**CTBP2 (tertile 3 vs. tertile 1)**	**0.004**	**0.69 (0.52–0.92)**	**0.010**	**<0.001**	**0.74 (0.56–0.98)**	**0.032**
DLL3 (tertile 3 vs. tertile 1)	0.004	0.84 (0.64–1.09)	0.189	0.003	0.73 (0.56–0.96)	0.022
**5. Oxidative stress response/Nrf2 (106 genes were analyzed)**						
ABCC3 (tertile 1 vs. tertile 3)	0.025	0.81 (0.62–1.06)	0.126	0.012	0.70 (0.53–0.92)	0.009
GSTA4 (tertile 3 vs. tertile 1)	0.010	0.75 (0.57–0.98)	0.035	0.034	0.81 (0.61–1.06)	0.120
**MAFF (tertile 1 vs. tertile 3)**	**0.010**	**0.74 (0.55–0.98)**	**0.036**	**0.032**	**0.64 (0.49–0.85)**	**0.002**
NQO1 (tertile 1 vs. tertile 3)	0.003	0.87 (0.66–1.15)	0.332	0.024	0.82 (0.62–1.07)	0.146
SLC2A10 (tertile 1 vs. tertile 3)	0.031	0.97 (0.73–1.28)	0.830	0.010	0.78 (0.59–1.04)	0.091
**SLC2A3 (tertile 1 vs. tertile 3)**	**0.001**	**0.75 (0.57–0.98)**	**0.037**	**0.008**	**0.71 (0.54–0.93)**	**0.012**
SLC2A9 (tertile 1 vs. tertile 3)	0.014	0.80 (0.61–1.06)	0.115	0.005	0.79 (0.60–1.05)	0.100
SLC6A13 (tertile 3 vs. tertile 1)	0.011	0.91 (0.69–1.21)	0.516	0.025	0.70 (0.53–0.93)	0.012
SQSTM1 (tertile 1 vs. tertile 3)	0.028	0.83 (0.63–1.09)	0.179	0.030	0.67 (0.51–0.88)	0.004
**6. PI3K signaling (97 genes were analyzed)**						
CSNK2B (tertile 3 vs. tertile 1)	0.010	0.70 (0.53–0.93)	0.013	0.011	0.81 (0.62–1.06)	0.121
CXCR4 (tertile 1 vs. tertile 3)	0.035	0.93 (0.71–1.22)	0.600	0.042	0.71 (0.54–0.95)	0.019
**ECSIT (tertile 2 vs. tertile 3)**	**0.004**	**1.69 (1.28–2.24)**	**<0.001**	**0.009**	**1.41 (1.07–1.86)**	**0.016**
**(tertile 2 vs. tertile 1)**	**0.004**	**1.56 (1.19–2.04)**	**0.001**	0.009	1.07 (0.82–1.40)	0.632
GNA14 (tertile 1 vs. tertile 3)	0.042	0.84 (0.64–1.11)	0.214	0.014	0.96 (0.73–1.25)	0.746
**HSP90B1 (tertile 1 vs. tertile 3)**	**0.005**	**0.74 (0.56–0.98)**	**0.034**	**0.001**	**0.57 (0.43–0.76)**	**<0.001**
MAPK8 (tertile 3 vs. tertile 1)	0.002	0.78 (0.60–1.03)	0.083	0.038	0.79 (0.60–1.04)	0.087
MYD88 (tertile 1 vs. tertile 3)	0.003	0.85 (0.64–1.12)	0.239	<0.001	0.67 (0.51–0.89)	0.005
RAC1 (tertile 1 vs. tertile 3)	0.019	1.00 (0.75–1.33)	0.996	0.003	0.72 (0.54–0.95)	0.018
SLA (tertile 1 vs. tertile 3)	0.001	0.81 (0.62–1.06)	0.124	0.001	0.71 (0.55–0.92)	0.010
SQSTM1 (tertile 1 vs. tertile 3)	0.028	0.83 (0.63–1.09)	0.179	0.030	0.67 (0.51–0.88)	0.004
**TNFRSF1A (tertile 1 vs. tertile 3)**	**0.003**	**0.75 (0.57–0.99)**	**0.041**	**<0.001**	**0.63 (0.48–0.83)**	**0.001**
VAV3 (tertile 1 vs. tertile 3)	0.028	0.78 (0.59–1.03)	0.078	0.018	0.74 (0.56–0.98)	0.038
**7. RTK signaling (80 genes were analyzed)**						
EIF4EBP1 (tertile 3 vs. tertile 1)	0.009	0.82 (0.63–1.09)	0.169	0.047	0.87 (0.67–1.14)	0.311
MAP2K2 (tertile 3 vs. tertile 1)	0.007	0.78 (0.60–1.03)	0.075	0.008	0.70 (0.54–0.91)	0.008
MAPK8 (tertile 3 vs. tertile 1)	0.002	0.78 (0.60–1.03)	0.083	0.038	0.79 (0.60–1.04)	0.087
**PAK1 (tertile 1 vs. tertile 3)**	**0.001**	**0.60 (0.45–0.80)**	**0.001**	**0.011**	**0.75 (0.57–0.99)**	**0.044**
**8. TGF-β signaling (79 genes were analyzed)**						
ACVR2B (tertile 3 vs. tertile 1)	0.022	0.85 (0.64–1.13)	0.261	<0.001	0.68 (0.52–0.90)	0.006
ID1 (tertile 3 vs. tertile 1)	0.049	0.77 (0.59–1.02)	0.069	0.023	0.82 (0.62–1.07)	0.140
**ID4 (tertile 2 vs. tertile 1)**	**0.040**	**1.53 (1.17–1.99)**	**0.002**	**0.006**	**1.41 (1.09–1.82)**	**0.009**
**(tertile 2 vs. tertile 3)**	**0.040**	**1.45 (1.10–1.91)**	**0.009**	**0.006**	**1.55 (1.18–2.04)**	**0.002**
**9. p53 signaling (59 genes were analyzed)**						
CDKN2A (tertile 3 vs. tertile 1)	0.002	0.79 (0.60–1.04)	0.093	0.015	0.78 (0.59–1.02)	0.072
**DDB2 (tertile 1 vs. tertile 3)**	**0.001**	**0.73 (0.55–0.97)**	**0.031**	**0.001**	**0.72 (0.54–0.96)**	**0.024**
FAS (tertile 1 vs. tertile 3)	0.042	0.97 (0.74–1.28)	0.824	0.004	0.75 (0.57–0.99)	0.043
IGFBP3 (tertile 1 vs. tertile 3)	0.001	0.76 (0.58–1.01)	0.055	<0.001	0.63 (0.48–0.83)	0.001
**MDM2 (tertile 1 vs. tertile 3)**	**<0.001**	**0.68 (0.52–0.90)**	**0.006**	**<0.001**	**0.63 (0.48–0.83)**	**0.001**
RPRM (tertile 3 vs. tertile 1)	0.024	0.77 (0.57–1.03)	0.074	0.028	0.73 (0.55–0.96)	0.024
SERPINE1 (tertile 1 vs. tertile 3)	0.006	0.83 (0.63–1.09)	0.173	0.003	0.71 (0.54–0.94)	0.016
STEAP3 (tertile 1 vs. tertile 3)	<0.001	0.83 (0.63–1.10)	0.192	<0.001	0.59 (0.44–0.77)	<0.001
TNFRSF10B (tertile 1 vs. tertile 3)	0.005	0.76 (0.58–1.01)	0.063	0.004	0.73 (0.55–0.96)	0.026
**10. Wnt/β-catenin signaling (31 genes were analyzed)**						
**DKK3 (tertile 1 vs. tertile 3)**	**<0.001**	**0.73 (0.55–0.97)**	**0.031**	**0.02**	**0.75 (0.56–0.99)**	**0.044**

GBM, glioblastoma multiforme; HR, hazard ratio; CI, confidence interval; CDC14A, cell division cycle 14A; CDKN2A, cyclin-dependent kinase inhibitor 2A; E2F2, E2F transcription factor 2; MAD2L1, mitotic arrest-deficient 2 like 1; MDM2, mouse double minute 2 homolog; SMC3, structural maintenance of chromosomes 3; ZBTB17, zinc finger and BTB domain-containing 17; AMOT, angiomotin; DLG5, discs large MAGUK scaffold protein 5; TIAL1, TIA1 cytotoxic granule-associated RNA binding protein like 1; WWTR1, WW domain-containing transcription regulator 1; CTBP2, C-terminal-binding protein 2; DLL3, delta-like canonical Notch ligand 3; Nrf2, nuclear factor erythroid 2-related factor 2; ABCC3, ATP binding cassette subfamily C member 3; GSTA4, glutathione S-transferase alpha 4; MAFF, MAF bZIP transcription factor F; NQO1, NAD(P)H quinone oxidoreductase 1; SLC2A10, solute carrier family 2 member 10; SLC2A3, solute carrier family 2 member 3; SLC2A9, solute carrier family 2 member 9; SLC6A13, solute carrier family 6 member 13; SQSTM1, sequestosome 1; PI3K, phosphatidylinositol 3-kinase; CSNK2B, casein kinase 2 beta; CXCR4, C-X-C motif chemokine receptor 4; ECSIT, evolutionarily conserved signaling intermediate in Toll pathways; GNA14, guanine nucleotide-binding protein subunit alpha-14; HSP90B1, heat shock protein 90 kDa beta member 1; MAPK8, mitogen-activated protein kinase 8; MYD88, myeloid differentiation primary response 88; RAC1, Ras-related C3 botulinum toxin substrate 1; SLA, Src-like adaptor; TNFRSF1A, tumor necrosis factor receptor superfamily member 1A; VAV3, Vav guanine nucleotide exchange factor 3; RTK, receptor tyrosine kinase; EIF4EBP1, eukaryotic translation initiation factor 4E-binding protein 1; MAP2K2, mitogen-activated protein kinase kinase 2; PAK1, p21 (RAC1) activated kinase 1; TGF, transforming growth factor beta 1; ACVR2B, activin A receptor type 2B; ID1, inhibitor of DNA binding 1; ID4, inhibitor of DNA binding 4; DDB2, damage-specific DNA-binding protein 2; FAS, Fas cell surface death receptor; IGFBP3, insulin-like growth factor-binding protein 3; RPRM, reprimo, TP53-dependent G2 arrest mediator homolog; SERPINE1, serpin family E member 1; STEAP3, STEAP3 metalloreductase; TNFRSF10B, tumor necrosis factor receptor superfamily member 10B; DKK3, dickkopf-3.

Tertile 1, 2, and 3 indicate low gene expression, moderate gene expression, and high gene expression, respectively.

The rows containing genes showing p < 0.05 in both overall survival and progression-free survival of multivariate Cox regression analyses are shown in bold.`

*Adjusted for sex, age, Karnofsky performance scale, radiation treatment, chemotherapy or immunotherapy, history of prior glioma.

For 8 of the above-mentioned genes—MAFF, SLC2A3, HSP90B1, TNFRSF1A, PAK1, DDB2, MDM2, and DKK3—being in the first tertile of gene expression was an independent predictor of better OS and PFS in GBM patients compared to the third tertile. Thus, higher expression of the above 8 genes was significantly associated with a higher risk of mortality and disease progression than the lower expression of those genes. In contrast, higher CTBP2 gene expression was significantly associated with better OS and PFS in GBM patients. However, E2F2 gene expression in the second tertile was associated with better OS and PFS than the genes in the first tertile in GBM patients. Moreover, the E2F2 expression in the second tertile of gene expression was associated with better OS than the genes in the highest tertile. In contrast, the second tertile of gene expression of ECSIT was associated with poor OS and PFS compared to those in the third tertile and poor OS compared to those in the first tertile. ID4 expression in the second tertile was also associated with poor OS and PFS compared to the genes whose expression was in the first and third tertiles. Therefore, moderate E2F2 gene expression and high or low ECSIT and ID4 gene expression was associated with a lower risk of mortality and disease progression in patients with GBM (except for disease progression at moderate E2F2 expression compared with high E2F2 expression and low ECSIT expression) (**Table 3**).

**Table 3:** Detailed information of the 12 genes from the 10 oncogenic signaling pathways, which are significantly associated with overall survival and progression-free survival in patients with GBM.

Gene symbol	Oncogenic signaling pathways	Gene expression levels showing better OS and PFS in patients with GBM	Summary of possible mechanisms of the 12 significant genes affecting OS and PFS in GBM patients	References
*E2F2*	Cell cycle signaling	Moderate expression (except when compared with high expression in PFS)	E2F2 is the center of the balance between cell proliferation and cell cycle arrest or apoptosis. Activation of deregulated E2F leading to both growth-promoting pathways or tumor suppressor pathways can result in oncogenic changes. If the amount of free E2F is below the threshold, E2F activates only growth-related target genes. However, when the amount of E2F exceeds the threshold, E2F activates not only growth-related targets but also pro-apoptotic targets.	(14, 15)
*CTBP2*	Notch signaling	High	CTBP2 is a nuclear transcriptional co-repressor. CTBP2 represses *Wnt* target genes, including β-catenin, thus leading to tumor suppression.	(16, 17)
*MAFF*	Nrf2 signaling	Low	Higher expression of MAFF promotes its binding to Nrf2, leading to increased expression of subsequent antioxidant enzymes. When stress conditions persist, MAFF and the Nrf2 complex activate ARE, leading to cell proliferation and tumorigenesis.	(18, 19)
*SLC2A3*		Low	*SLC2A3* encodes the GLUT3. Because of excessively high glucose consumption of tumor cells, aberrant *GLUT* family expression, including *GLUT3*, is known to associate with poor prognosis in various cancers, including brain tumors.	(20–22)
*ECSIT*	PI3K signaling	High or low (except for low expression in PFS)	A TRAF6-ECSIT complex is crucial for the generation of mROS *via* Toll-like receptors. ECSIT also regulates the production of mROS.	(23)
*HSP90B1*		Low	Protein complex, containing PTN, SPARC, SPARCL1, and HSP90B, facilitates the migration of glioma cells.	(24, 25)
*TNFRSF1A*		Low	*TNFRSF1A* encodes the TNFα receptor. TNFα and IL6 induce sustained NF-κB activity, aberrant activation of STAT3, and increased expression of pro-oncogenic proteins. The cross-talk between NF-κB and STAT3 induces tumor progression and facilitates cancer stemness in gliomas.	(26, 27)
*PAK1*	RTK signaling	Low	PAK1 functions as a node for multiple signaling pathways. *PAK1* overexpression is associated with activation of PI3K/AKT/mTOR and facilitates cross-talk between the Ras effector pathways and the Wnt signaling pathway associated with tumor progression, migration, and angiogenesis.	(28, 29)
*ID4*	TGF-β signaling	High or low	ID4 can act as a tumor suppressor and an oncogene in different tumor types. ID4 may act as a metastatic suppressor and inhibits the aggressive invasive behavior of GBM. At the same time, however, ID4 also promotes angiogenesis in GBM.	(30–33)
*DDB2*	p53 signaling	Low	DDB2 is known as a sensor of DNA damage that plays a critical role in DNA repair system. However, in cancer cell, DDB2 may also promotes repair of cancer DNA lesions induced by radiation or chemotherapy, leading to chemo/radioresistance.	(34–36)
*MDM2*	p53 signaling, cell cycle	Low	The transcription factor p53 plays critical roles in the suppression of tumor development. MDM2 is the primary negative regulatory factor of the p53 protein.	(37)
*DKK3*	Wnt/β-catenin signaling	Low	DKK3 may modulate cancer cell malignant potentials by activating AKT thorough the binding of DKK3 to RTK or Wnt receptors and by intracellular protein-protein interactions of DKK3b.	(4, 38)

GBM, glioblastoma multiforme; OS, overall survival; PFS, progression-free survival; E2F2, E2F transcription factor 2; CTBP2, C-terminal-binding protein 2; MAFF, MAF bZIP transcription factor F; Nrf2, nuclear factor erythroid 2-related factor 2; ARE, antioxidant response element; SLC2A3, solute carrier family 2 member 3; GLUT3, glucose transporter, type 3; PI3K, phosphatidylinositol 3-kinase; TRAF6, tumor necrosis factor receptor associated factor 6; ECSIT, evolutionarily conserved signaling intermediate in Toll pathways; mROS, mitochondrial reactive oxygen species; HSP90B1, heat shock protein 90 kDa beta member 1; PTN, pleiotrophin; SPARC, secreted protein acidic and cysteine rich; SPARCL1, SPARC like 1; TNFRSF1A, tumor necrosis factor receptor superfamily member 1A; TNF, tumor necrosis factor; IL, interleukin; NF-κB, nuclear factor-kappa B; STAT3, signal transducer and activator of transcription; PAK1, p21 activated kinase 1; AKT, protein kinase B; mTOR, mammalian target of rapamycin; RTK, receptor tyrosine kinase; ID4, inhibitor of DNA binding 4; TGF-β, transforming growth factor beta; DDB2, damage-specific DNA-binding protein 2; MDM2, mouse double minute 2 homolog; DKK3, dickkopf-3.

### Possible mechanisms of the 12 genes affecting OS and PFS in GBM patients

**Figure 2:**
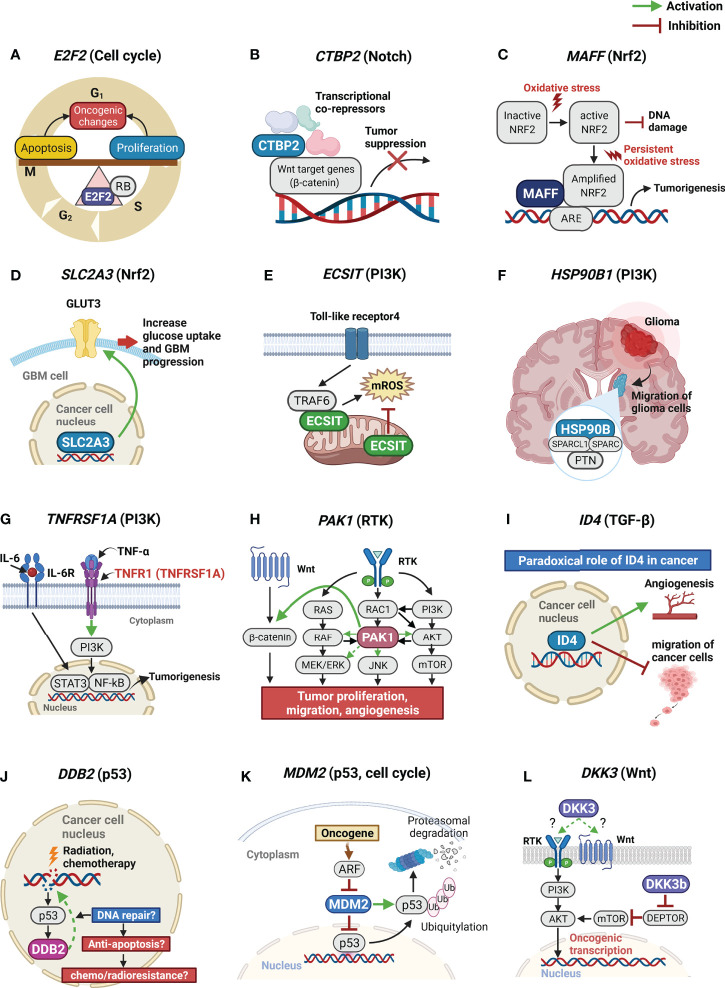
Schematic illustrations of possible roles of the 12 significant genes in GBM. GBM, glioblastoma. **(A)** E2F2 is the center of the balance between cell proliferation and cell cycle arrest or apoptosis; **(B)** CTBP2 is a nuclear transcriptional co-repressor. CTBP2 represses *Wnt* target genes leading to tumor suppression; **(C)** Higher expression of MAFF promotes its binding to Nrf2. When stress conditions persist, MAFF and the Nrf2 complex activate ARE, leading to tumorigenesis; **(D)** SLC2A3 encodes the GLUT3. Because of excessively high glucose consumption of tumor cells, aberrant GLUT3 expression is known to associate with poor prognosis in brain tumors; **(E)** A TRAF6-ECSIT complex is crucial for the generation of mROS. ECSIT also regulates the production of mROS; **(F)** Protein complex, containing PTN, SPARC, SPARCL1, and HSP90B, facilitates the migration of glioma cells; **(G)** *TNFRSF1A* encodes the TNFα receptor. TNFα and IL6 induce sustained NF-κB activity, aberrant activation of STAT3, and increased expression of pro-oncogenic proteins; **(H)** PAK1 functions as a node for multiple signaling pathways. *PAK1* overexpression is associated with activation of PI3K/AKT/mTOR and facilitates cross-talk between the Ras effector pathways and the Wnt signaling pathway associated with tumor progression, migration, and angiogenesis; **(I)** ID4 can act as a tumor suppressor and an oncogene in different tumor types. ID4 may act as a migration suppressor of GBM. At the same time, however, ID4 also promotes angiogenesis in GBM. **(J)** DDB2 plays a critical role in DNA repair system. However, in cancer cell, DDB2 may also promotes repair of cancer DNA lesions induced by radiation or chemotherapy, leading to chemo/radioresistance. **(K)** MDM2 is the primary negative regulatory factor of the p53 protein; **(L)** DKK3 may modulate cancer cell malignant potentials by activating AKT thorough the binding of DKK3 to RTK or Wnt receptors and by intracellular protein-protein interactions of DKK3b. GBM, glioblastoma multiforme; E2F2, E2F transcription factor 2; CTBP2, C-terminal-binding protein 2; MAFF, MAF bZIP transcription factor F; Nrf2, nuclear factor erythroid 2-related factor 2; ARE, antioxidant response element; SLC2A3, solute carrier family 2 member 3; GLUT3, glucose transporter, type 3; TRAF6, tumor necrosis factor receptor associated factor 6; ECSIT, evolutionarily conserved signaling intermediate in Toll pathways; mROS, mitochondrial reactive oxygen species; PTN, pleiotrophin; SPARC, secreted protein acidic and cysteine rich; SPARCL1, SPARC like 1; HSP90B1, heat shock protein 90 kDa beta member 1; TNFRSF1A, tumor necrosis factor receptor superfamily member 1A; TNF, tumor necrosis factor; IL, interleukin; NF-κB, nuclear factor-kappa B; STAT3, signal transducer and activator of transcription; PAK1, p21 activated kinase 1; PI3K, phosphatidylinositol 3-kinase; AKT, protein kinase B; mTOR, mammalian target of rapamycin; ID4, inhibitor of DNA binding 4; DDB2, damage-specific DNA-binding protein 2; MDM2, mouse double minute 2 homolog; DKK3, dickkopf-3; RTK, receptor tyrosine kinase.

Based on our study findings, we show schematic illustrations of the possible roles of the 12 significant genes in GBM in **Figure 2**. We also present a table summarizing our findings and possible mechanisms of the 12 significant genes affecting OS and PFS in GBM patients (**Table 3**).

### Correlations between the expression of the 12 genes and between the expression of the 12 genes and CD8+ T cells in GBM tissues

**Figure 3:**
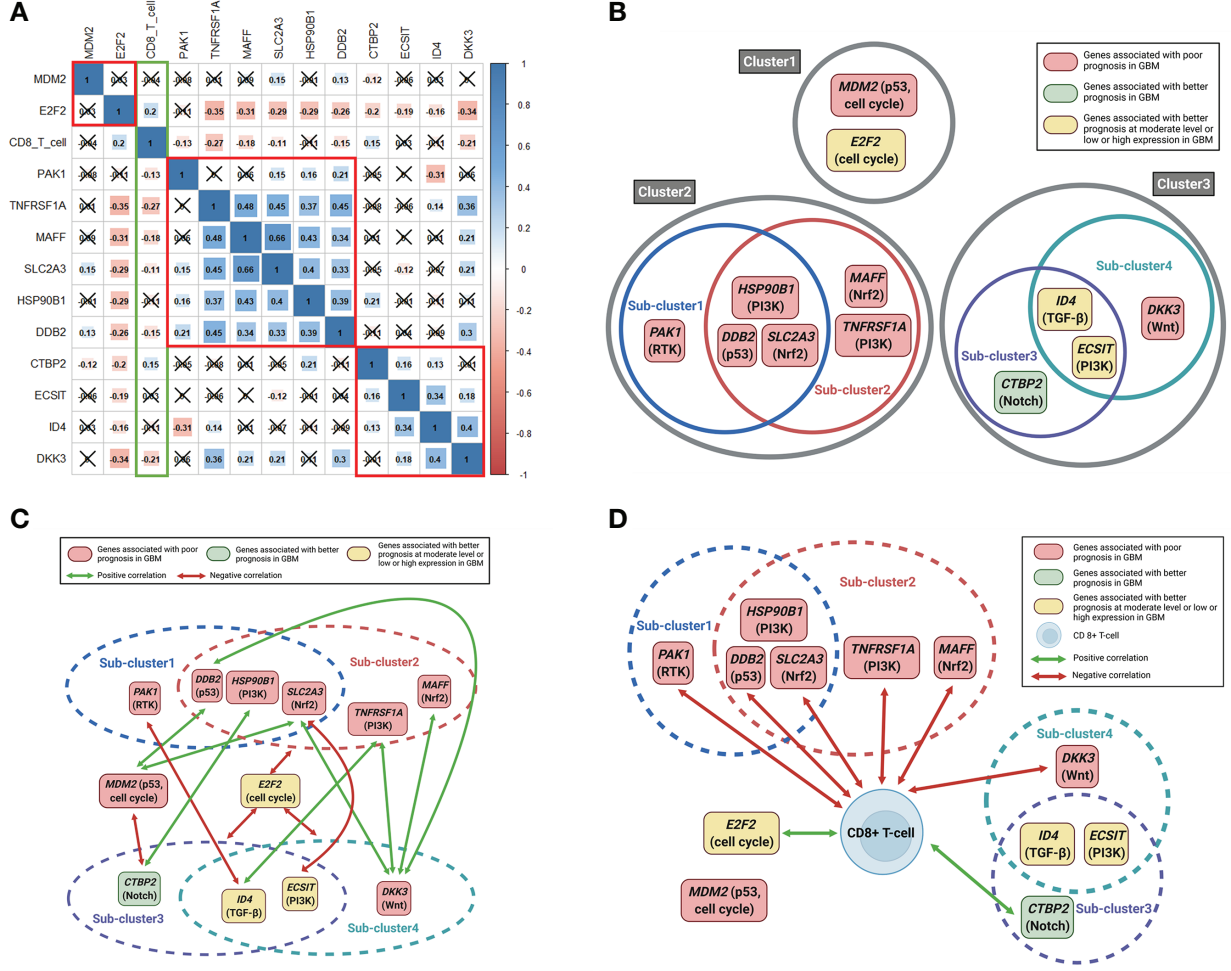
Correlation plots between 12 significant genes and between 12 significant genes and CD8+ T-cell fractions. Schematic illustrations of correlations between the 12 significant genes and between the 12 significant genes and CD8+ T-cell fractions. **(A)** Pearson correlation coefficients and significance levels were calculated between the 12 significant genes and between the 12 significant genes and CD8+ T-cell fractions. The color-coordinated legend indicates the value and sign of Pearson’s correlation coefficient. The number in the box indicates Pearson’s correlation coefficient. Moreover, an x in the box indicates a p value ≥ 0.001; **(B)** the 12 significant genes are largely classified into three clusters according to the significance of the correlation between mutual genes. Clusters 2 and 3 were again divided into two sub-clusters; **(C)** correlations between gene expression outside the clusters or subclusters; **(D)** correlations between the expression of the 12 significant genes and CD8+ T cell fractions.

When we estimated the correlations between the mRNA expression of the 12 above-mentioned genes, we observed that the 12 genes are largely divided into three clusters according to the significance of the correlation between the expression of these genes (**Figures 3A, B**). Clusters 2 and 3 were again divided into two sub-clusters based on the significance of the correlation between the expression of the genes (**Figures 3A, B**). Sub-cluster 1 consists of PAK1, HSP90B1, DDB2, and SLC2A3; sub-cluster 2 consists of HSP90B1, DDB2, SLC2A3, MAFF, and TNFRSF1A; sub-cluster 3 consists of CTBP2, ID4, and ECSIT; and sub-cluster 4 consists of ID4, ECSIT, and DKK3. When we identified the correlations between gene expression outside the clusters or subclusters, we observed that E2F2 had negative correlations with all genes of sub-clusters 2, 3, and 4 (**Figure 3C**). In addition, the expression of both MDM2 and DKK3 showed positive correlations with DDB2 and SLC2A3 expression, while DKK3 expression also showed positive correlations with TNFRSF1A and MAFF expression. Other correlations between the expression of these genes are shown in **Figure 3C**. We then estimated the correlations between the expression of the 12 genes and CD8+ T cell fractions. The expression of most of the genes associated with poor prognosis in GBM such as PAK1, DDB2, SLC2A3, TNFRSF1A, MAFF, and DKK3 showed significant, negative correlations with CD8+ T immune cell infiltrations in GBM tissues (**Figures 3A, D**). We also observed positive correlations of CD8+ T-cell fractions with CTBP2 and E2F2 expression, which were associated with a better prognosis in GBM at high and moderate levels of expression, respectively (**Figure 3D**).

### Identification of antitumor agents for the suppression of genes related to poor prognosis in GBM patients

**Figure 4:**
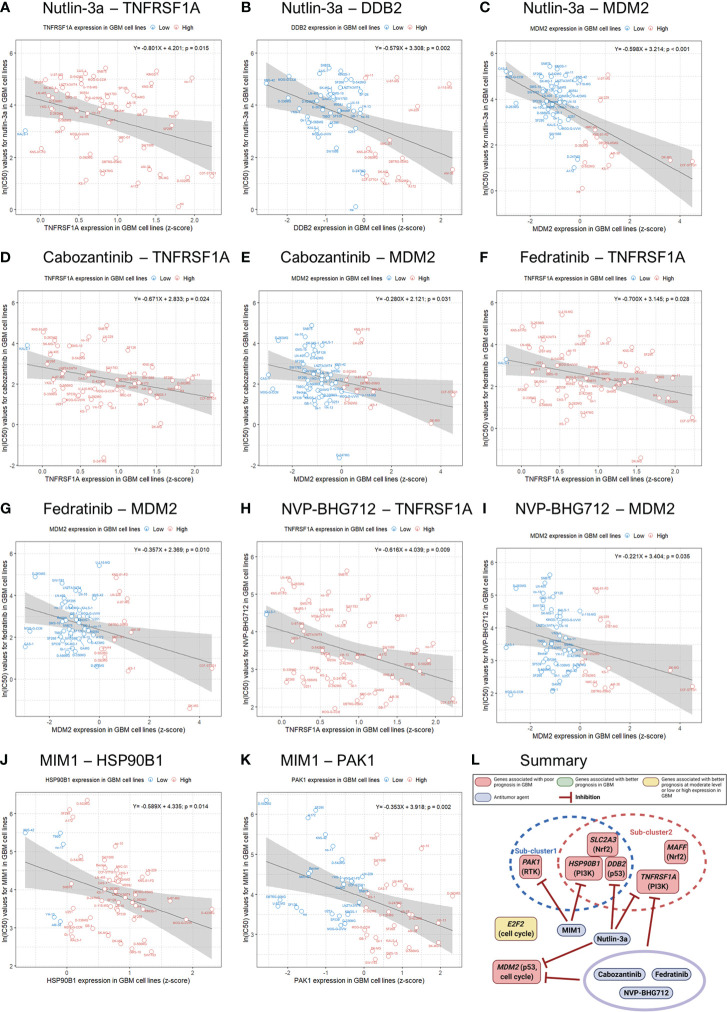
Genomics of Drug Sensitivity in Cancer (GDSC) database analysis and an illustration showing actions of antitumor agents on several significant genes associated with poor prognosis in GBM patients. Linear regression analysis shows associations between gene expression and the natural log of the half-maximal inhibitory concentration [ln(IC50)] values for antitumor agents in 49 GBM cell lines (blue, low gene expression; red, high gene expression).

We evaluated the correlations and significance levels between gene expression and ln(IC50) values for 316 antitumor drugs in 49 GBM cell lines. A negative correlation between gene expression and the ln(IC50) value for an antitumor agent indicates that the antitumor agent effectively inhibits the expression of the corresponding gene. Therefore, we identified antitumor agents effective against the genes related to poor prognosis in GBM, showing negative correlations between gene expression and ln(IC50) values with p-values less than 0.05 (**Table 4**). Antitumor agents that effectively inhibited the expression of at least two identified significant genes in GBM cell lines were nutlin-3a, cabozantinib, fedratinib, NVP-BHG712, and MIM1 (**Figure 4**). Nutlin-3a effectively inhibited DDB2, TNFRSF1A, and MDM2 expression. In addition, cabozantinib, fedratinib, and NVP-BHG712 also effectively inhibited both TNFRSF1A and MDM2 expression, while MIM1 effectively blocked PAK1 and HSP90B1 expression (**Figure 4**).

**Table 4:** Identification of antitumor agents for the genes significantly associated with poor prognosis in GBM.

Gene symbol	Antitumor agents	Pearson’s correlation coefficient between gene expression and ln(IC50) values for antitumor agents in 49 GBM cell lines	p
MAFF	Serdemetan	-0.431	0.002
	Pictilisib	-0.405	0.005
	Allitinib (AST-1306)	-0.340	0.017
	Devimistat (CPI-613)	-0.332	0.020
	Gefitinib	-0.324	0.023
	AZD6482	-0.322	0.024
	IMD-0354	-0.314	0.028
	BX795	-0.308	0.032
	LGK974	-0.296	0.039
	PFI-3	-0.313	0.044
	KIN001-042	-0.289	0.044
	AZD8055	-0.294	0.045
	Pictilisib	-0.293	0.045
	JQ12	-0.289	0.047
	Wnt-C59	-0.282	0.049
SLC2A3	Not available in the GDSC and COSMIC databases		
HSP90B1	LDN-193189	-0.391	0.005
	Olaparib	-0.375	0.009
	Bleomycin	-0.356	0.012
	Mcl-1 inhibitor molecule 1 (MIM1)	-0.349	0.014
	5Z-7-Oxozeaenol	-0.346	0.015
	Bosutinib	-0.329	0.021
	Avagacestat	-0.325	0.023
	BAX activator, molecule 7 (BAM7)	-0.322	0.024
	ICL1100013	-0.324	0.025
	Cytarabine	-0.309	0.031
	Refametinib	-0.304	0.038
	Piperlongumine	-0.296	0.039
	Afatinib	-0.288	0.045
	PD184352 (CI-1040)	-0.286	0.046
TNFRSF1A	Ponatinib	-0.484	<0.001
	Foretinib	-0.448	0.001
	NVP-BHG712	-0.367	0.009
	Nutlin-3a	-0.345	0.015
	Pazopanib	-0.345	0.020
	Cabozantinib	-0.323	0.024
	Fedratinib	-0.314	0.028
	TPCA-1	-0.282	0.049
PAK1	LCL161	-0.470	0.001
	Mcl-1 inhibitor molecule 1 (MIM1)	-0.423	0.002
	ZG-10	-0.516	0.006
	AT7867	-0.386	0.006
	GSK429286A	-0.377	0.008
	CD532	-0.369	0.009
	XMD8-92	-0.484	0.012
	Flavopiridol	-0.325	0.023
	WZ-1-84	-0.475	0.025
	GSK269962A	-0.452	0.027
	AGI-6780	-0.305	0.033
	Panobinostat	-0.302	0.035
	QL-VIII-58	-0.389	0.045
	Procaspase-Activating Compound 1 (PAC-1)	-0.299	0.046
	Trichostatin A	-0.286	0.047
	Z-VAD-FMK	-0.285	0.050
DDB2	Nutlin-3a	-0.427	0.002
MDM2	Nutlin-3a	-0.590	<0.001
	JW-7-24-1	-0.396	0.005
	Fedratinib	-0.366	0.010
	JNJ-38877605	-0.312	0.029
	Quizartinib	-0.311	0.030
	Cabozantinib	-0.308	0.031
	NVP-BHG712	-0.302	0.035
	MPS-1-IN-1	-0.300	0.036
	XMD14-99	-0.286	0.046
DKK3	Tipifarnib	-0.389	0.007
	Navitoclax	-0.338	0.018

GBM, glioblastoma multiforme; ln(IC50), natural log of the half-maximal inhibitory concentration; CTBP2, C-terminal-binding protein 2; MAFF, MAF bZIP transcription factor F; SLC2A3, solute carrier family 2 member 3; HSP90B1, heat shock protein 90 kDa beta member 1; TNFRSF1A, tumor necrosis factor receptor superfamily member 1A; PAK1, p21-activated kinase 1; DDB2, damage-specific DNA-binding protein 2; MDM2, mouse double minute 2 homolog; DKK3, dickkopf-3.

## Discussion

We recently used TCGA data to report that DKK3, a component of the Wnt/β-catenin signaling pathway, is associated with higher mortality, disease progression, and chemoresistance in patients with GBM (4). To expand the scope of our study, we identified genes whose expression was significantly associated with mortality and disease progression in GBM patients in TCGA from all genes belonging to well-known 10 canonical oncogenic pathways, including Wnt/β-catenin signaling (3). We then observed that there were 12 genes whose expression was significantly related to both mortality and disease progression in patients with GBM. These 12 genes were E2F2 (cell cycle signaling pathway), CTBP2 (Notch signaling), MAFF (Nrf2 signaling), SLC2A3 (Nrf2 signaling), ECSIT (PI3K signaling), HSP90B1 (PI3K signaling), TNFRSF1A (PI3K signaling), PAK1 (RTK signaling), ID4 (TGF-β signaling), DDB2 (p53 signaling), MDM2 (p53 and cell cycle signaling), and DKK3 (Wnt/β-catenin signaling). To the best of our knowledge, this study is the first to identify these 12 genes belonging to 10 oncogenic pathways that significantly affect both mortality and disease progression in GBM patients.

Considering the role of these 12 genes in cancer, we briefly suggest possible mechanisms affecting the prognosis of GBM as follows (1). E2F2 is known as the center of the balance between cell proliferation and apoptosis (14). As shown in **Figure 3C** of our study, E2F2 was negatively correlated with all 10 significant genes in subcluster 2-4 (except for PAK1 and MDM2). Therefore, we hypothesized that E2F2 may be a control tower regulating tumor aggressiveness in GBM. However, the activation of deregulated E2F leading to both growth-promoting pathways or tumor suppressor pathways can result in oncogenic changes (14). In addition, if the level of free E2F is below the threshold, E2F activates only growth-related target genes. When the level of E2F exceeds the threshold, E2F activates not only growth-related targets but also pro-apoptotic targets (14, 15). Therefore, we hypothesized that E2F2 must be maintained at appropriate levels to properly regulate cell proliferation and apoptosis. Any pathological increase or decrease in the E2F2 levels can adversely affect the prognosis of GBM (2). CTBP2 is known as a nuclear transcriptional co-repressor, repressing Wnt target genes and thus leading to tumor suppression (16, 17). According to **Figures 3C, D** CTBP2 expression is negatively correlated with MDM2 expression, which is the primary negative regulatory factor of p53, but is positively associated with CD8+ T-cell fractions. Therefore, we believe that high CTBP2 expression is associated with a better prognosis in GBM (3). MAFF binds to Nrf2 and leads to the increased expression of subsequent antioxidant enzymes. When stress conditions persist, MAFF and the Nrf2 complex activate the antioxidant response element (ARE), leading to cell proliferation and tumorigenesis (18, 19) (4). SLC2A3 encodes the glucose transporter 3 (GLUT3) (20). Because of the excessively high glucose consumption of brain tumor cells, GLUT3 is associated with a poor prognosis in GBM (20–22) (5). ECSIT forms a complex with TRAF6 in mitochondria to generate mitochondrial reactive oxygen species (mROS). However, ECSIT also inhibits mROS in mitochondria (23). Therefore, ECSIT performs two seemingly contradictory roles in mitochondria. Although the exact mechanism is unknown, according to our results, the prognosis of GBM was better when ECSIT expression was increased or decreased than moderate level (6). It is known that the protein complex of HSP90B with PTN, SPARC, and SPARCL1 facilitates the migration of glioma cells (7, 24, 25). TNFRSF1A encodes the TNFα receptor (39). TNFα and IL6 induce sustained NF-κB activity and aberrant activation of STAT3. The cross-talk between NF-κB and STAT3 induces tumor progression and facilitates cancer stemness in gliomas (8, 26, 27). PAK1 functions as a node for multiple signaling pathways. PAK1 overexpression is associated with the activation of the PI3K/AKT/mTOR signaling pathway (28). In addition, PAK1 facilitates the cross-talk between the Ras effector pathways and the Wnt signaling pathway that are associated with tumor progression, migration, and angiogenesis (29). Therefore, we believe that the increase of interactions of PAK1 with multiple oncogenic signaling pathways adversely affects the prognosis of patients with GBM in this study (9). ID4 has a paradoxical role in cancer. ID4 may act as a metastatic suppressor and inhibit the aggressive invasive behavior of GBM (30, 31). At the same time, however, ID4 also promotes angiogenesis in GBM (32, 33). We believe that this paradoxical role of ID4 might be associated with a better prognosis in GBM patients when ID4 expression was high or low than moderate level in our study (10). DDB2 is known as a sensor of DNA damage that plays a critical role in the DNA repair system. However, DDB2 is reported to have dual functions in cancer, sometimes with tumor suppressive properties and sometimes functioning as an oncogene (34). In cancer cells, DDB2 may also promote the repair of cancer DNA lesions induced by radiation or chemotherapy leading to chemo/radioresistance (34–36) (11). The transcription factor p53 plays critical roles in the suppression of tumor development, and MDM2 is the primary negative regulatory factor of p53 (37). Therefore, we believe that MDM2 was significantly associated with a poor prognosis in GBM patients in this study (12). DKK3 may be associated with GBM aggressiveness by activating AKT through the binding of DKK3 to RTK or Wnt receptors and by intracellular protein-protein interactions of DKK3b (4, 38). According to **Figure 3C**, DKK3 expression was positively correlated with the expression of genes associated with a poor prognosis in GBM such as DDB2, SLC2A3, TNFRSF1A, and MAFF. We recently reported that DKK3 expression is associated with GBM immunosuppression (4). In this study, we also observed that the expression of six of the genes significantly associated with a poor prognosis of GBM, showed negative correlations with CD8+ T-cell fractions in GBM tissue (**Figure 3D**). The immunosuppressive tumor microenvironment of GBM is known to lead to a poor prognosis in patients with GBM (40). It is noteworthy that most of the genes associated with a poor prognosis of GBM in this study, were also related to immunosuppressive conditions in GBM.

We used GDSC data to investigate drug sensitivity in 49 GBM cell lines according to the several genes significantly associated with a poor prognosis in GBM. Although, there were many antitumor agents inhibiting the expression of these genes, nutlin-3a, cabozantinib, fedratinib, NVP-BHG712, and Mcl-1 inhibitor molecule 1 (MIM1) effectively and concurrently inhibited the expression of at least two significant genes in GBM cell lines. Nutlin-3a, cabozantinib, fedratinib, and NVP-BHG712 effectively blocked the expression of both TNFRSF1A and MDM2 in GBM cell lines. To our knowledge, nutlin-3a and cabozantinib have been reported to be effective against GBM, but there have been no reports of the efficacy of fedratinib and NVP-BHG712 against GBM (41, 42). Meanwhile, MIM1 effectively blocked HSP90B1 and PAK1 expression in GBM cell lines. Previously, MIM1 was reported to induce apoptosis and sensitize GBM cells to alkylating agents (43). Unfortunately, we were not able to identify drugs that inhibit SLC2A3 expression because there was no information on SLC2A3 expression in 49 GBM cell lines in the GDSC and COSMIC databases. We also expect to develop antitumor agents that simultaneously block the expression of more genes related to the poor prognosis of GBM.

Our study has some limitations. First, as this study is retrospective in nature and based on TCGA data, further prospective studies are needed to validate the results. However, because we used TCGA public data, researchers can check and verify our results. Second, there were no experimental analyses to investigate the relationships between the 12 above-mentioned genes and GBM cells, and further in vitro and/or in vivo studies are necessary. Third, missing data and gene sets associated with 10 oncogenic signaling pathways that were not available in TCGA data may affect the results of statistical analysis in the study. Last, the relationships between significant genes and mortality and disease progression classified by GBM molecular subtypes were not analyzed.

In conclusion, despite these limitations, we used a large-scale, open database to identify 12 genes whose expression was significantly related to mortality and disease progression of GBM among genes belonging to 10 well-known oncogenic canonical pathways. We present the possible mechanisms by which these 12 genes affect both mortality and disease progression of GBM. We also identified that most of these genes were related to immunosuppressive conditions in GBM. Nutlin-3a, cabozantinib, fedratinib, NVP-BHG712, and MIM1 effectively and concurrently inhibited the expression of at least two of these genes in GBM cell lines. We believe that our findings will contribute to improve the understanding of the mechanisms underlying GBM pathophysiology and help develop treatments for patients with GBM in the future.

## Data availability statement

The datasets presented in this study can be found in online repositories. The names of the repository/repositories and accession number(s) can be found in the article/**Supplementary Material**.

## Author contributions

Conception and design: MHH. Data acquisition: MHH, KWM. Data analysis/interpretation: MHH. Visualization: MHH. Supervision and critical review of the manuscript: All. Final approval of submission: All.

## Funding

Y-KN was partly supported by NRF/MSIT (No. 2018R1A5A7059549, 2021M3E5D2A01019545), IITP/MSIT Artifcial Intelligence Graduate School Program for Hanyang University (2020-0-01373).

## Acknowledgments

Illustrations created with BioRender.

## Conflict of interest

The authors declare that the research was conducted in the absence of any commercial or financial relationships that could be construed as a potential conflict of interest.

## Publisher’s note

All claims expressed in this article are solely those of the authors and do not necessarily represent those of their affiliated organizations, or those of the publisher, the editors and the reviewers. Any product that may be evaluated in this article, or claim that may be made by its manufacturer, is not guaranteed or endorsed by the publisher.
