# Supplementary material for: Identification of genes from ten oncogenic pathways associated with mortality and disease progression in glioblastoma
Source: Front Oncol. 2022 Aug 10;12:965638. doi: 10.3389/fonc.2022.965638 (PMC9399757; doi:10.3389/fonc.2022.965638)
Supplement: Supplementary file 1 [file DataSheet_1.docx]

**Supplementary Material**

Identification of genes from ten oncogenic pathways associated with mortality and disease progression in glioblastoma

Myung-Hoon Han, Kyueng-Whan Min, Yung-Kyun Noh, Jae Min Kim, Jin Hwan Cheong, Je Il Ryu, Yu Deok Won, Seong-Ho Koh, Young Mi Park


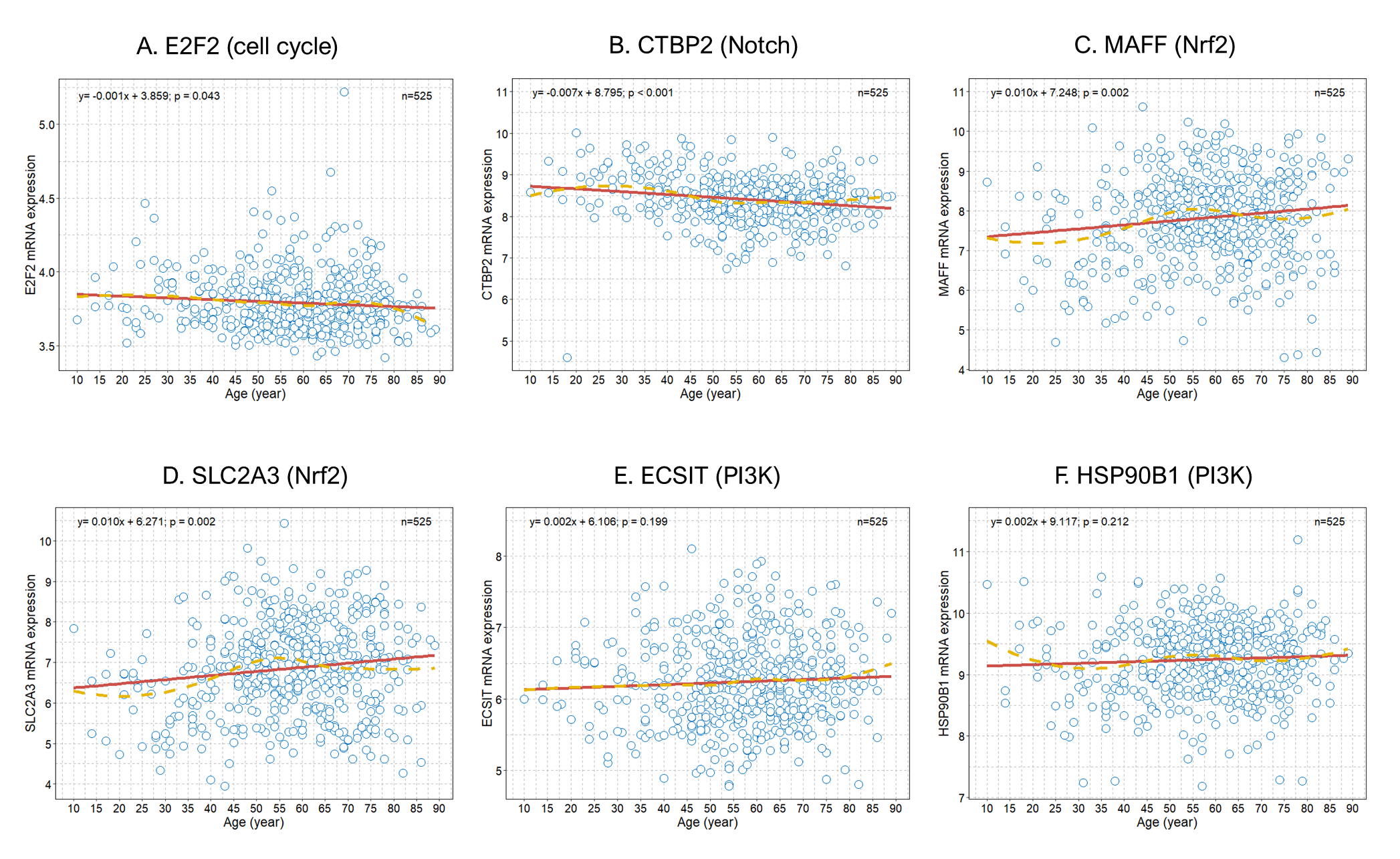


**Supplementary Figure 1.** Scatterplots with linear regression lines (red) and LOWESS curves (yellow) show correlations between age and E2F2, CTBP2, MAFF, SLC2A3, ECSIT, and HSP90B1 gene expressions in patients with GBM.

LOWESS, locally weighted scatter plot smoothing; E2F transcription factor 2; CTBP2, C-terminal-binding protein 2; MAFF, MAF bZIP transcription factor F; SLC2A3, solute carrier family 2 member 3; ECSIT, evolutionarily conserved signaling intermediate in Toll pathways; HSP90B1, heat shock protein 90 kDa beta member 1; GBM, glioblastoma.


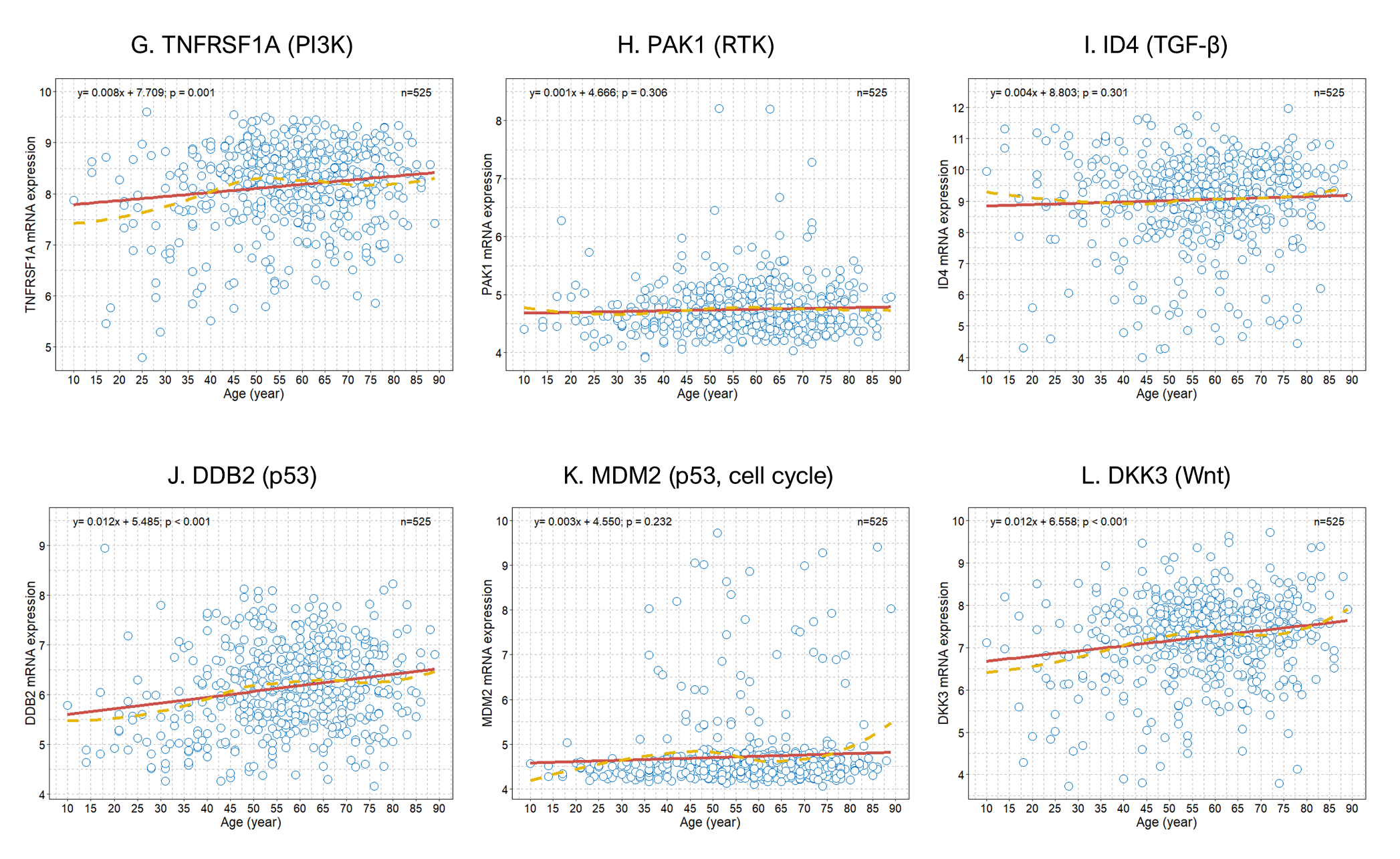


**Supplementary Figure 2.** Scatterplots with linear regression lines (red) and LOWESS curves (yellow) show correlations between age and TNFRSF1A, PAK1, ID4, DDB2, MDM2, and DKK3 gene expressions in patients with GBM.

LOWESS, locally weighted scatter plot smoothing; TNFRSF1A, tumor necrosis factor receptor superfamily member 1A; PAK1, p21 activated kinase 1; ID4, inhibitor of DNA binding 4; DDB2, damage-specific DNA-binding protein 2; MDM2, mouse double-minute 2 homolog; DKK3, dickkopf-3; GBM, glioblastoma.

**Supplementary Table 1.** Differences between the radiation treatment population and the adjuvant chemotherapy and/or immunotherapy population on the basis of expression of 12 selected genes in patients with GBM.

|  | Radiation treatment (+) (n=435) | | |  | Adjuvant chemotherapy and/or immunotherapy (+)  (n=205) | | |
| --- | --- | --- | --- | --- | --- | --- | --- |
| Selected 12 genes | n | percent | p |  | n | percent | p |
| 1. E2F2 |  |  | 0.550 |  |  |  | 0.252 |
| Tertile 1 | 140 | 80.0 |  |  | 77 | 44.0 |  |
| Tertile 2 | 150 | 85.7 |  |  | 65 | 37.1 |  |
| Tertile 3 | 145 | 82.9 |  |  | 63 | 36.0 |  |
| 2. CTBP2 |  |  | 0.723 |  |  |  | 0.780 |
| Tertile 1 | 142 | 81.1 |  |  | 66 | 37.7 |  |
| Tertile 2 | 147 | 84.0 |  |  | 72 | 41.1 |  |
| Tertile 3 | 146 | 83.4 |  |  | 67 | 38.3 |  |
| 3. MAFF |  |  | 0.294 |  |  |  | **0.045** |
| Tertile 1 | 148 | 84.6 |  |  | 65 | 37.1 |  |
| Tertile 2 | 146 | 83.4 |  |  | 59 | 33.7 |  |
| Tertile 3 | 141 | 80.6 |  |  | 81 | 46.3 |  |
| 4. SLC2A3 |  |  | 0.971 |  |  |  | 0.408 |
| Tertile 1 | 147 | 84.0 |  |  | 67 | 38.3 |  |
| Tertile 2 | 143 | 81.7 |  |  | 63 | 36.0 |  |
| Tertile 3 | 145 | 82.9 |  |  | 75 | 42.9 |  |
| 5. ECSIT |  |  | 0.820 |  |  |  | **0.034** |
| Tertile 1 | 145 | 82.9 |  |  | 61 | 34.9 |  |
| Tertile 2 | 142 | 81.1 |  |  | 62 | 35.4 |  |
| Tertile 3 | 148 | 84.6 |  |  | 82 | 46.9 |  |
| 6. HSP90B1 |  |  | 0.686 |  |  |  | 0.208 |
| Tertile 1 | 147 | 84.0 |  |  | 59 | 33.7 |  |
| Tertile 2 | 149 | 85.1 |  |  | 73 | 41.7 |  |
| Tertile 3 | 139 | 79.4 |  |  | 73 | 41.7 |  |
| 7. TNFRSF1A |  |  | 0.624 |  |  |  | 0.585 |
| Tertile 1 | 146 | 83.4 |  |  | 70 | 40.0 |  |
| Tertile 2 | 139 | 79.4 |  |  | 63 | 36.0 |  |
| Tertile 3 | 150 | 85.7 |  |  | 72 | 41.1 |  |
| 8. PAK1 |  |  | 0.693 |  |  |  | 0.329 |
| Tertile 1 | 150 | 85.7 |  |  | 63 | 36.0 |  |
| Tertile 2 | 143 | 81.7 |  |  | 66 | 37.7 |  |
| Tertile 3 | 142 | 81.1 |  |  | 76 | 43.4 |  |
| 9. ID4 |  |  | 0.467 |  |  |  | 0.329 |
| Tertile 1 | 145 | 82.9 |  |  | 66 | 37.7 |  |
| Tertile 2 | 146 | 83.4 |  |  | 63 | 36.0 |  |
| Tertile 3 | 144 | 82.3 |  |  | 76 | 43.4 |  |
| 10. DDB2 |  |  | 0.717 |  |  |  | 0.614 |
| Tertile 1 | 145 | 82.9 |  |  | 68 | 38.9 |  |
| Tertile 2 | 150 | 85.7 |  |  | 73 | 41.7 |  |
| Tertile 3 | 140 | 80.0 |  |  | 64 | 36.6 |  |
| 11. MDM2 |  |  | 0.055 |  |  |  | 0.968 |
| Tertile 1 | 155 | 88.6 |  |  | 67 | 38.3 |  |
| Tertile 2 | 144 | 82.3 |  |  | 69 | 39.4 |  |
| Tertile 3 | 136 | 77.7 |  |  | 69 | 39.4 |  |
| 10. DKK3 |  |  | 0.163 |  |  |  | 0.064 |
| Tertile 1 | 152 | 86.9 |  |  | 59 | 33.7 |  |
| Tertile 2 | 143 | 81.7 |  |  | 66 | 37.7 |  |
| Tertile 3 | 140 | 80.0 |  |  | 80 | 45.7 |  |

GBM, glioblastoma; E2F transcription factor 2; CTBP2, C-terminal-binding protein 2; MAFF, MAF bZIP transcription factor F; SLC2A3, solute carrier family 2 member 3; ECSIT, evolutionarily conserved signaling intermediate in Toll pathways; HSP90B1, heat shock protein 90 kDa beta member 1; TNFRSF1A, tumor necrosis factor receptor superfamily member 1A; PAK1, p21 activated kinase 1; ID4, inhibitor of DNA binding 4; DDB2, damage-specific DNA-binding protein 2; MDM2, mouse double-minute 2 homolog; DKK3, dickkopf-3.
